# Dual functions: A coumarin–chalcone conjugate inhibits cyclic‐di‐GMP and quorum‐sensing signaling to reduce biofilm formation and virulence of pathogens

**DOI:** 10.1002/mlf2.12087

**Published:** 2023-09-24

**Authors:** Yu Zhang, Pramod Bhasme, Dinesh S. Reddy, Dejian Liu, Zhaoxiao Yu, Tianhu Zhao, Yaqian Zheng, Amit Kumar, Haiying Yu, Luyan Z. Ma

**Affiliations:** ^1^ State Key Laboratory of Microbial Resources, Institute of Microbiology Chinese Academy of Sciences Beijing China; ^2^ University of Chinese Academy of Sciences Beijing China; ^3^ Centre for Nano and Material Sciences Jain University Bangalore Karnataka India

**Keywords:** biofilm, cyclic‐di‐GMP, exopolysaccharide Psl, *Pseudomonas aeruginosa*, quorum sensing

## Abstract

Antibiotic resistance or tolerance of pathogens is one of the most serious global public health threats. Bacteria in biofilms show extreme tolerance to almost all antibiotic classes. Thus, use of antibiofilm drugs without bacterial‐killing effects is one of the strategies to combat antibiotic tolerance. In this study, we discovered a coumarin–chalcone conjugate C9, which can inhibit the biofilm formation of three common pathogens that cause nosocomial infections, namely, *Pseudomonas aeruginosa*, *Staphylococcus aureus*, and *Escherichia coli*, with the best antibiofilm activity against *P. aeruginosa*. Further investigations indicate that C9 decreases the synthesis of the key biofilm matrix exopolysaccharide Psl and bacterial second messenger cyclic‐di‐GMP. Meanwhile, C9 can interfere with the regulation of the quorum sensing (QS) system to reduce the virulence of *P. aeruginosa*. C9 treatment enhances the sensitivity of biofilm to several antibiotics and reduces the survival rate of *P. aeruginosa* under starvation or oxidative stress conditions, indicating its excellent potential for use as an antibiofilm‐forming and anti‐QS drug.

## Impact statement

Bacteria in biofilms show extreme tolerance to almost all traditional antibiotics; hence, it is critical to find new strategies or drugs to combat biofilms. We report a novel antibiofilm compound, coumarin–chalcone conjugate C9, which inhibits biofilm formation by targeting the biofilm matrix exopolysaccharide and intracellular signaling molecular cyclic‐di‐GMP. C9 can sensitize the bacteria in biofilms to antibiotics with little bacterium‐killing activity unless under starvation or oxidative stress conditions. Moreover, C9 alleviates the virulence of *Pseudomonas aeruginosa* and reduces the survival rate of this bacterium, indicating its anti‐inflammation potential. Overall, our study provides a novel strategy to combat biofilm‐related complications.

## INTRODUCTION

Biofilms are communities of microorganisms enmeshed in a self‐secreted extracellular matrix^1^, ^2^. More than 65% of human infections are related to bacterial biofilms. Bacteria in biofilms show extreme tolerance to almost all traditional antibiotics and are also protected from the host immune clearance^3^; they are approximately 1000 times more resistant to antibiotics than their planktonic counterparts^4^, ^5^. The extracellular polymeric substance (EPS) is considered to maintain the biofilm architecture and functions as a matrix holding biofilm cells together and protecting them. Thus, the EPS matrix plays a very critical role in the biofilm resistance phenotype. However, little has been reported about compounds that can target the EPS matrix to improve the clearance of biofilms.

Nosocomial infections are threats to the health of patients with compromised immune systems^6^. The most common pathogens that cause nosocomial infections include Gram‐negative bacteria *Pseudomonas aeruginosa* and *Escherichia coli* and Gram‐positive bacteria *Staphylococcus aureus*, all of which can form biofilms and are key priority pathogens listed by the World Health Organization for research and development of new antibiotics^7^, ^8^. Decreasing the virulence of pathogens is a highly optimal strategy to control nosocomial infections. Therefore, a new avenue of drug discovery in recent years has been to seek compounds or reagents that have antibiofilm activity and/or can reduce the virulence of pathogens.

*P. aeruginosa* is an opportunistic pathogen that survives in a wide variety of environments. This bacterium is well known for its ability to infect people with immunodeficiency and cause many diseases such as pneumonia, lung infection in cystic fibrosis patients, and chronic obstructive pulmonary diseases^3^, ^4^. Over the last 25 years, the incidence of both community‐acquired and hospital‐acquired *P. aeruginosa* infections has increased^9^. One of the major reasons for this is the ability of *P. aeruginosa* to form biofilms, which results in increased costs of hospitalizations^10^. *P. aeruginosa* has become a model organism for biofilm research and has been well studied^11^. Three exopolysaccharides, Psl, Pel, and alginate, are involved in the biofilm formation of *P. aeruginosa*. As a key scaffolding matrix component of the *P. aeruginosa* biofilm, Psl forms a fiber‐like matrix of bacterial communities and also confers resistance to antibiotics and phagocytic cells^11^. Cyclic‐di‐GMP (c‐di‐GMP) is an important second messenger controlling a wide range of cellular processes in many bacteria, such as exopolysaccharide biosynthesis, bacterial motility, and biofilm formation^12^, ^13^. In general, a high level of c‐di‐GMP promotes biofilm formation, whereas a lower level promotes motility^14^. The intracellular c‐di‐GMP level is determined by the expression of diguanylate cyclases (DGCs) and phosphodiesterases (PDEs), which catalyze the biosynthesis and degradation of c‐di‐GMP molecules. *P. aeruginosa* contains approximately 40 genes encoding DGCs and/or PDEs^15^, which can be potential targets for the design or discovery of new compounds or drugs to combat biofilms.

The success of *P. aeruginosa* infections relies not only on the ability of *P. aeruginosa* to form biofilms but also on its production of virulence factors. Quorum sensing (QS) regulates gene expression by means of population sensing, through which bacteria communicate with each other by secreting small diffusible signaling molecules to control the expression of virulent factors and the growth of biofilms^16^, ^17^, ^18^. In *P. aeruginosa*, a large number of genes, including many genes encoding virulence factors and genes that are involved in biofilm development, are activated by two interconnected QS systems, LasR‐LasI and RhlR‐Rhl, which are responsible for the production of QS signaling molecules, N‐(3‐oxododecanoyl) homoserine lactone (3‐oxo‐C_12_‐HSL) and N‐butyryl homoserine lactone (C_4_‐HSL)^19^. Other QS signaling molecules have also been identified and classified into the 4‐quinolone family^19^. *P. aeruginosa* produces 2‐heptyl‐4‐quinolone (HHQ) and 2‐heptyl‐3‐hydroxy‐4‐quinolone (PQS), which are better known as *Pseudomonas* PQS signaling molecules^20^, ^21^. Both HHQ and PQS regulate the expression of several genes via the PqsR (also named as MvfR)^19^, ^20^, ^22^. PQS and C_4_‐HSL both stimulate the production of pyocyanin, a virulent factor that itself has also signaling activity^23^, ^24^. Remarkably, the QS system of *P. aeruginosa* is required for its full virulence in various hosts, including zebrafish, fruitflies, nematodes, and mice (burn wound, pneumonia, and chronic lung infection models)^25^. As a consequence, significant research efforts have been undertaken to identify and use QS‐interfering agents to control *P. aeruginosa*‐related infections.

Chalcones (1,3‐diaryl‐2‐propen‐1‐ones) are one of the important classes of naturally available compounds with a widespread distribution in plants, fruits, spices, vegetables, tea, and soy‐based foodstuff. The very basic scaffolds of chalcones promote the synthesis of chalcone derivatives^26^. Previous studies have indicated the promising biological activities of both naturally occurring and synthetic chalcone derivatives against microbe and cancer hallmarks by targeting signaling molecules or key elements, providing valuable insights for the potential use of chalcone derivatives as therapeutic agents^27^, ^28^. Coumarins (2H‐1‐benzopyran‐2‐ones) are another important class of naturally occurring compounds that have potential therapeutic properties for bacteria‐related infections^29^, ^30^. They form a huge class of important lactones with a linked structure of benzene and an α‐pyrone ring and have a π–π conjugated system rich in electrons and with excellent charge transport properties^31^. This unique structure of coumarins enables their derivatives to readily interact with diverse enzymes and receptors in organisms through weak bond interactions, and hence coumarins have excellent potential for use as medicinal drugs^31^. The ability of coumarins to block QS signaling systems and to inhibit biofilm formation in plants as well as clinically relevant pathogens has been highlighted in several recent reports^32^, ^33^. Thereby, from a synthetic perspective, the potential biological significance of chalcones and coumarins intrigued us to hypothesize that the additive of the coumarin and chalcones conjugate might produce a synergistic effect in enhancing their bio‐activity. We thus focused on utilizing the scaffolds of chalcones and coumarins as conjugates. In this study, we synthesized coumarin–chalcone conjugated compounds (C1–C12) and tested their antibiofilm activity. C9 showed the best antibiofilm activity, with no interference on bacterial growth under nutrient‐sufficient conditions. In addition, it significantly reduced the virulence of *P. aeruginosa*. We also further determined the action of C9 at the molecular level. Our results indicated that C9 has excellent potential for use as an antibiofilm and anti‐QS drug.

## RESULTS

### Coumarin–chalcone conjugate C9 has the potential to be a broad‐spectrum antibiofilm drug

**Figure 1:**
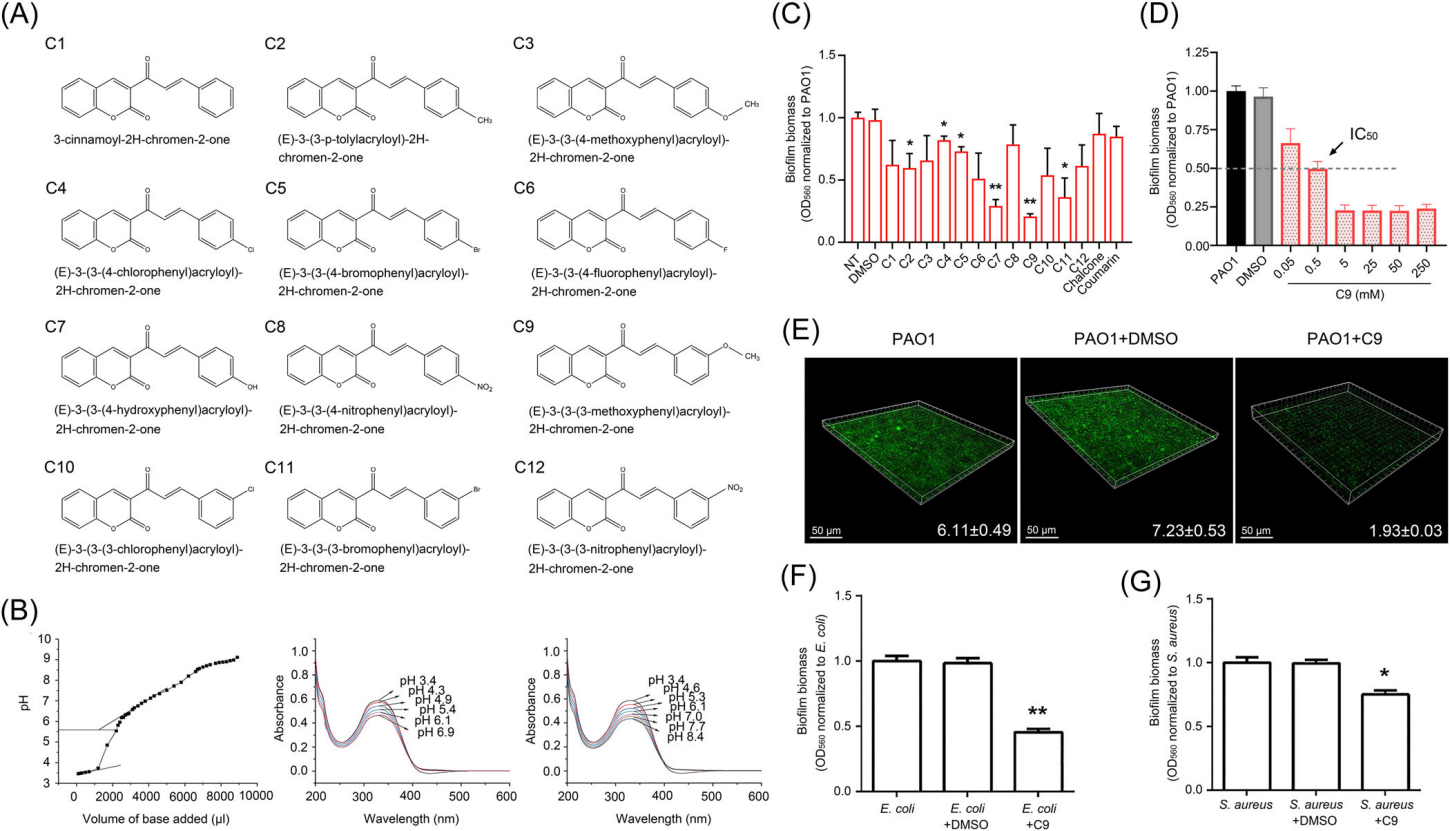
Coumarin–chalcone conjugates decreases biofilm formation of *Pseudomonas aeruginosa*. (A) Structures of synthesized coumarin–chalcone conjugates (C1–C12). (B) Stability analysis of C9. Left panel: pH titration curve of C9 obtained by plotting pH versus the volume of KOH added; middle panel: titration of C9 with HCl; right panel: titration of compound C9 with KOH. (C) Quantification of PAO1 biofilms in the presence of coumarin or chalcone or conjugates C1–C12. Biofilms were quantified after 24 h of incubation and are represented as adhesion units (OD_560_). OD_560_ was normalized to the level of PAO1 without treatment (the biofilm biomass of PAO1 is 7.82). (D) Effect of different concentrations of C9 on the biofilm formation of PAO1. (E) Confocal laser scanning microscopy (CLSM) images of *P. aeruginosa* biofilms. The biofilms were treated with compound C9 or DMSO alone and stained with SYTO9 (green). The value of biofilm biomass (μm^3^/μm^2^) is shown in the corresponding images. (F) Quantification of *S. aureus* biofilm biomass in the presence of C9. (G) Quantification of *E. coli* biofilm biomass in the presence of C9. Error bars indicate standard deviations; significance was determined using Student's *t*‐test compared with controls (*p < 0.05, **p < 0.01, *S. aureus* biofilm biomass is 4.7 and *E. coli* biofilm biomass is 5.8).

The structures of the synthesized coumarin–chalcone conjugates C1–C12 are shown in Figure 1A, and the structure of the compounds was confirmed by the NMR spectrum (Figure S1). The physicochemical properties of compounds, such as biological membrane permeability and lipophilicity, are significantly affected by their pKa value. A graph of pH versus the volume of KOH added was plotted to determine the equivalence point and thereby pKa (Figure 1B). The pKa value for C9 was found to be 5.66, which indicated that the compound had the ability to cross the lipid membrane barrier and enter the target cells^34^. It is also important to determine whether the synthesized compounds are stable at a range of pH values since our body pH varies from acidic to basic. Therefore, we used UV–visible spectral analysis to study the stability of C9 at pH values ranging from 3.4 to 8.4. First, HCl (25 mM) was added continuously to C9 (initial pH 6.9) until the pH reached 3.4 (Figure 1B), resulting in an increased intensity of the absorption band at 330 nm. Then, the pH value was gradually increased to 8.4 by titrating with KOH (25 mM) (Figure 1B). The reversibility of the reaction indicates that protonation occurs during titration with HCl. It was also clear that the absorbance bands maintained their positions throughout the experiments, no additional bands were detected, and the original absorbance bands remained intact, which indicates that C9 is stable at a range of pH values.

We then evaluated the effect of C1–C12 on biofilm formation of the *P. aeruginosa* PAO1 strain using a crystal violet assay. Among 12 compounds, C9, C7, and C11 significantly decreased the biofilm biomass of PAO1 by 70%, 60%, and 55%, respectively, while the other compounds showed a moderate effect (Figure 1C). Therefore, C9 was selected for characterization as well as further determination of its inhibitory effect on pellicle formation of PAO1. The confocal laser scanning microscopy (CLSM) analysis (Figure 1E) indicated that C9 could reduce the pellicle formation of PAO1 by 73%. The biofilm biomass of PAO1 decreased in a C9 concentration‐dependent manner (Figure 1D). The IC_50_ of C9 (the concentration that can inhibit 50% of biofilm biomass) was ~0.5 and 5 mM C9 showed the strongest inhibitory effect on biofilm formation, which exerted a similar inhibitory effect as that of  25–250 mM C9 (Figure 1D). However, the concentration of 5 mM C9 could not disrupt a formed biofilm (Figure S2B). The addition of C9 did not have a significant impact on the growth of PAO1 (Figure S2E) or T4P‐mediated twitching as well as flagella‐mediated swimming motility (Figure S2C,D), indicating that the biofilm‐inhibitory ability of C9 may not be due to its effect on bacterial growth or motilities. Furthermore, the antibiofilm efficacy of C9 against other bacteria was also examined. C9 significantly inhibited the biofilm formation of Gram‐positive pathogen *S. aureus* (Figure 1G) as well as another Gram‐negative bacterium *E. coli* (Figure 1F). It showed better activity on *E. coli* biofilm (60% reduction) than *S. aureus* biofilm (30% reduction). Similar to PAO1, the growth of *E. coli* and *S. aureus* was not affected by C9 (Figure S2E). Altogether, our data indicated that C9 had the potential for use as a broad‐spectrum antibiofilm‐forming drug.

### Coumarin–chalcone conjugate C9 inhibits the biofilm formation of *P. aeruginosa* by decreasing exopolysaccharide Psl production and the intracellular c‐di‐GMP level

**Figure 2:**
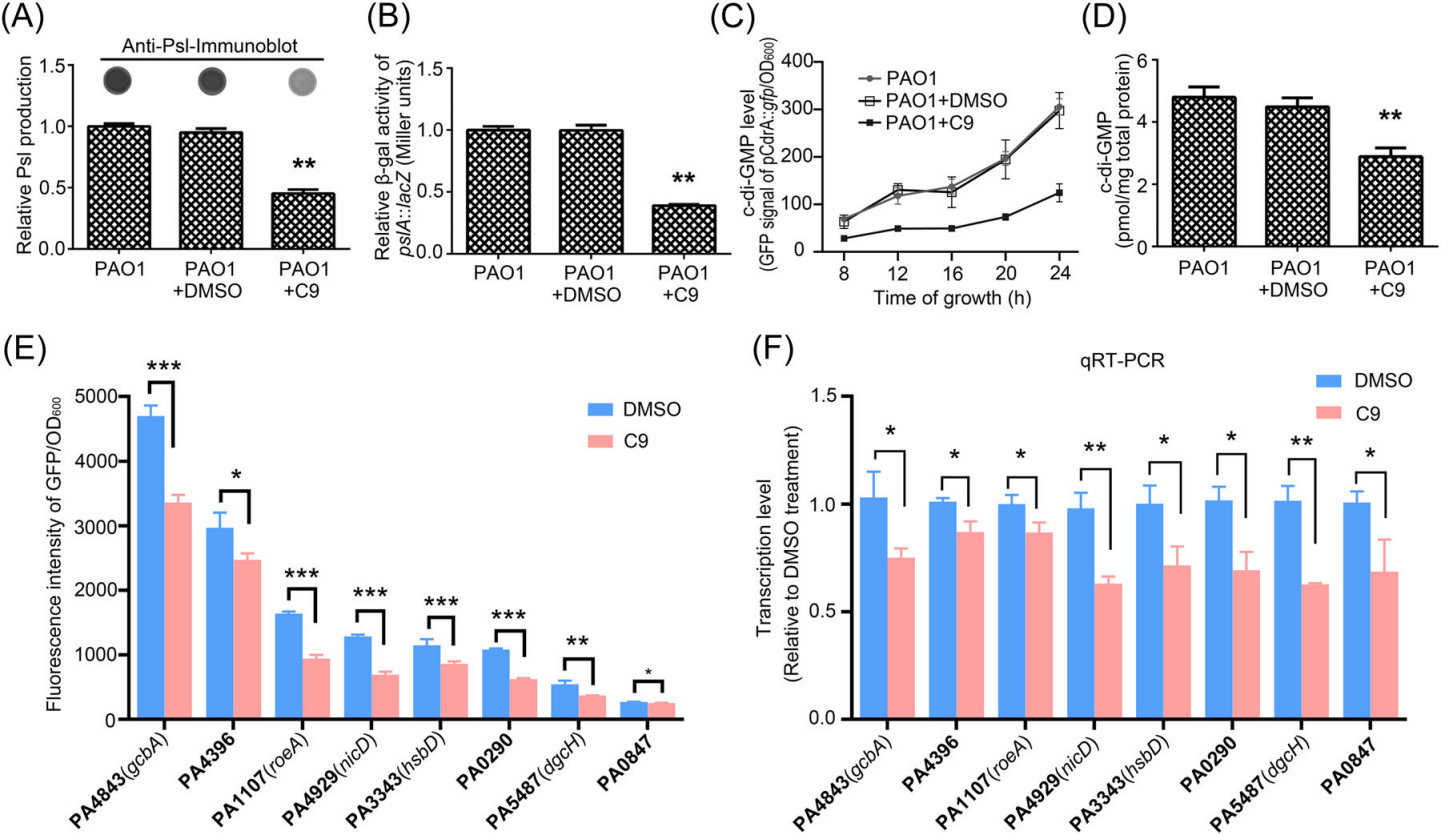
Effect of C9 on Psl production and c‐di‐GMP. (A) Psl was measured using the anti‐Psl immunoblotting technique. The value was normalized to the level of PAO1 (Psl production of PAO1 is 31 µg/OD_600_). (B) Transcriptional *lacZ* fusion construct assayed for β‐galactosidase activities showed downregulation of *pslA* mediated by compound C9. The value was normalized to the no‐treatment control (β‐galactosidase activity is 232/OD_415_). (C) The c‐di‐GMP levels in all cultures were indicated by the green fluorescent intensity of plasmid pCdrA::*gfp* ^s^ during 24 h of growth. (D) Intracellular c‐di‐GMP concentration measured by LC‐MS/MS. (E, F) Effects of C9 on the transcription of genes involved in the synthesis of c‐di‐GMP by the green fluorescent intensity of the GFP‐tagged promoter region in the pPROBE‐AT′ vector and qRT‐PCR. Error bars indicate standard deviations; significance was determined using Student's *t*‐test compared with controls (*p < 0.05, **p < 0.01, ***p < 0.001). LC‐MS, liquid chromatography–mass spectrometry.

Considering the critical role of exopolysaccharide Psl in the biofilm formation of *P. aeruginosa*, we examined the effect of C9 on the production of Psl. Our results showed that C9 reduced 60% of Psl production (Figure 2A), and the analysis of the *pslA*::*lacZ* transcriptional fusion reporter indicated that C9 decreased the transcription of the *psl* operon to reduce Psl production (Figure 2B). The intracellular c‐di‐GMP is an important signaling molecule to control biofilm formation. We then tested the effect of C9 on the intracellular concentration of c‐di‐GMP in *P. aeruginosa* by using both an in vivo method and an in vitro measurement. The indirect in vivo measurement used the CdrA::*gfp* ^s^ reporter as previously published^35^. The results showed that C9 significantly decreased the fluorescent intensity of PAO1/pCdrA::*gfp* ^s^ (Figure 2C), indicating that C9 reduced the synthesis of c‐di‐GMP. c‐di‐GMP from the PAO1 strain grown with or without C9 was also directly extracted, followed by liquid chromatography–mass spectrometry (LC‐MS)/MS quantification (Figure 2D). A 2‐fold reduction of the c‐di‐GMP concentration was induced by C9 treatment, which is in accordance with the above results of the pCdrA::*gfp* ^s^ reporter. We further investigated the transcription of 17 genes that have been reported or predicted to encode DGCs in *P. aeruginosa* to synthesize c‐di‐GMP^36^. Out of the 17 genes, transcription of eight genes, namely, PA4843 (*gcbA*), PA4396, PA1107 (*roeA*), PA4929 (*nicD*), PA3343 (*hsbD*), PA0290, PA5487 (*dgcH*), and PA0847, was significantly reduced by C9 (Figure 2E,F). Taken together, these results indicated that the addition of C9 decreased the transcription of genes involved in the synthesis of exopolysaccharide Psl and c‐di‐GMP in *P. aeruginosa*, leading to the reduction of Psl and c‐di‐GMP synthesis, which can thus efficiently inhibit biofilm formation.

### C9 downregulates the expression of QS genes and decreases *P. aeruginosa* virulence

**Figure 3:**
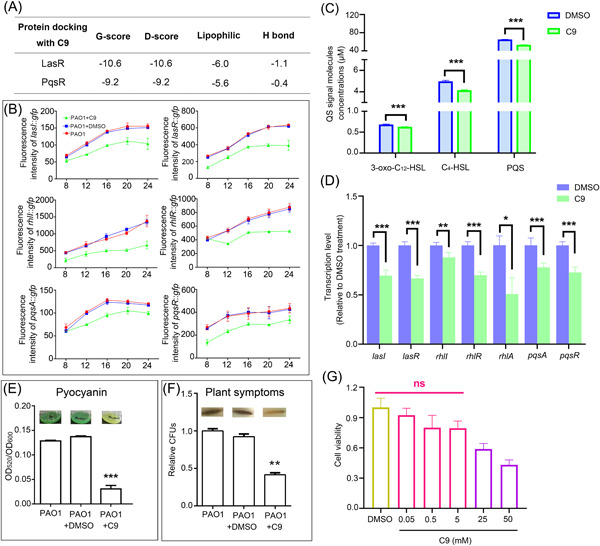
Inhibition of QS systems in *Pseudomonas aeruginosa* by compound C9. (A) Results of docking analysis. The binding energies (kcal/mol) of C9 with *lasR* and *pqsR* were tabulated along with the interacting hydrogen‐bonding residues. (B) Effect of C9 on the transcription of QS‐related genes (*lasI, lasR*, *rhII, rhIR, rhlA, pqsA*, and *pqsR*) by the green fluorescent intensity of the GFP‐tagged promoter region in the pPROBE‐AT′ vector. (C) Quantification of 3‐oxo‐C12‐HSL (DMSO: 0.7 ± 0.01 μM, C9: 0.6 ± 0.01 μM), C4‐HSL (DMSO: 5.0 ± 0.09 μM, C9: 4.1 ± 0.05 μM), and PQS (DMSO: 65.0 ± 0.2 μM, C9: 53.3 ± 0.2 μM) by LC‐MS/MS. (D) Transcriptional changes of QS‐related genes from PAO1 with or without C9 treatments by qRT‐PCR. (E) Effect of C9 on pyocyanin production. (F) Effect of C9 on the virulence of *P. aeruginosa* PAO1 examined in Chinese cabbage leaves. A representative picture of infected midribs is also shown for each treatment. The relative number of bacterial cells (as colony‐forming units, CFUs) presented in per mg of cabbage midrib 36 h postinjection is shown. The value was normalized to the PAO1 no‐treatment control (3.69 × 10^7^ CFU/mg). (G) Cytotoxicity of C9 on macrophages. Error bars indicate standard deviations; significance was determined using Student's *t*‐test compared with PAO1 (*p < 0.05; **p < 0.01; ***p < 0.001; ns, no significance). DMSO, dimethyl sulfoxide; qRT‐PCR, quantitative reverse‐transcription‐PCR; QS, quorum sensing.

QS systems regulate biofilm formation and the expression of many virulent factors. Molecular docking analysis indicated that C9 could strongly bind to LasR and PqsR, which are two key QS regulators in *P. aeruginosa* (Figure S3). The binding energies (kcal/mol) of C9 with LasR and PqsR from the docking analysis are shown in Figure 3A. We thus further investigated the impact of C9 on the QS system. The LC‐MS/MS quantification showed that the production of three QS signal molecules (3‐oxo‐C_12_‐HSL, C_4_‐HSL, and PQS) was significantly decreased by the addition of C9 (Figure 3C). In addition, both GFP transcriptional fusion reporters and qRT‐PCR were used to examine the expression level of QS genes. The transcription of four LasR‐activated genes, namely, *lasI, lasR*, *rhlI*, and *rhlA*, was reduced by C9 (Figure 3B,D). Moreover, the expression of *pqsR* and *pqsA* (*pqsA* is the first gene of the *pqs* operon, which encodes enzymes for the synthesis of PQS signaling molecules) was also downregulated by the addition of C9 (Figure 3B,D). These results indicated that C9 repressed QS regulation, likely by inhibiting the activity of LasR and PqsR.

Many virulent factors are activated by the QS system, we thus hypothesize that C9 treatment could reduce the virulence of *P. aeruginosa*. We first examined the effect of C9 on the production of pyocyanin, an important virulent factor in *P. aeruginosa*, whose expression was regulated by both LasR and PqsR. Consistently, the treatment of C9 inhibited the production of pyocyanin by more than 75% (Figure 3E). We then further carried out an in vivo experiment to test the impact of C9 on the virulence of *P. aeruginosa* by using the Chinese cabbage infection model. In this model, results were analyzed by observing phenotypes and then counting the CFUs in the infected regions. The results showed that C9 decreased *P. aeruginosa* virulence in Chinese cabbage by more than 60% (Figure 3F). Both results were consistent with the above changes in QS regulation induced by C9, indicating that C9 is an effective anti‐QS and antivirulence compound. We also evaluated the cytotoxicity of C9 on macrophages. The results indicated that 5 mM C9 had minimal effect on the viability of macrophages (Figure 3G), further indicating the potential of C9 for use as an antibiofilm and anti‐QS drug.

### C9 treatment sensitizes bacteria in biofilms to antibiotics and reduces their survival rates under starvation and oxidative stress conditions

**Figure 4:**
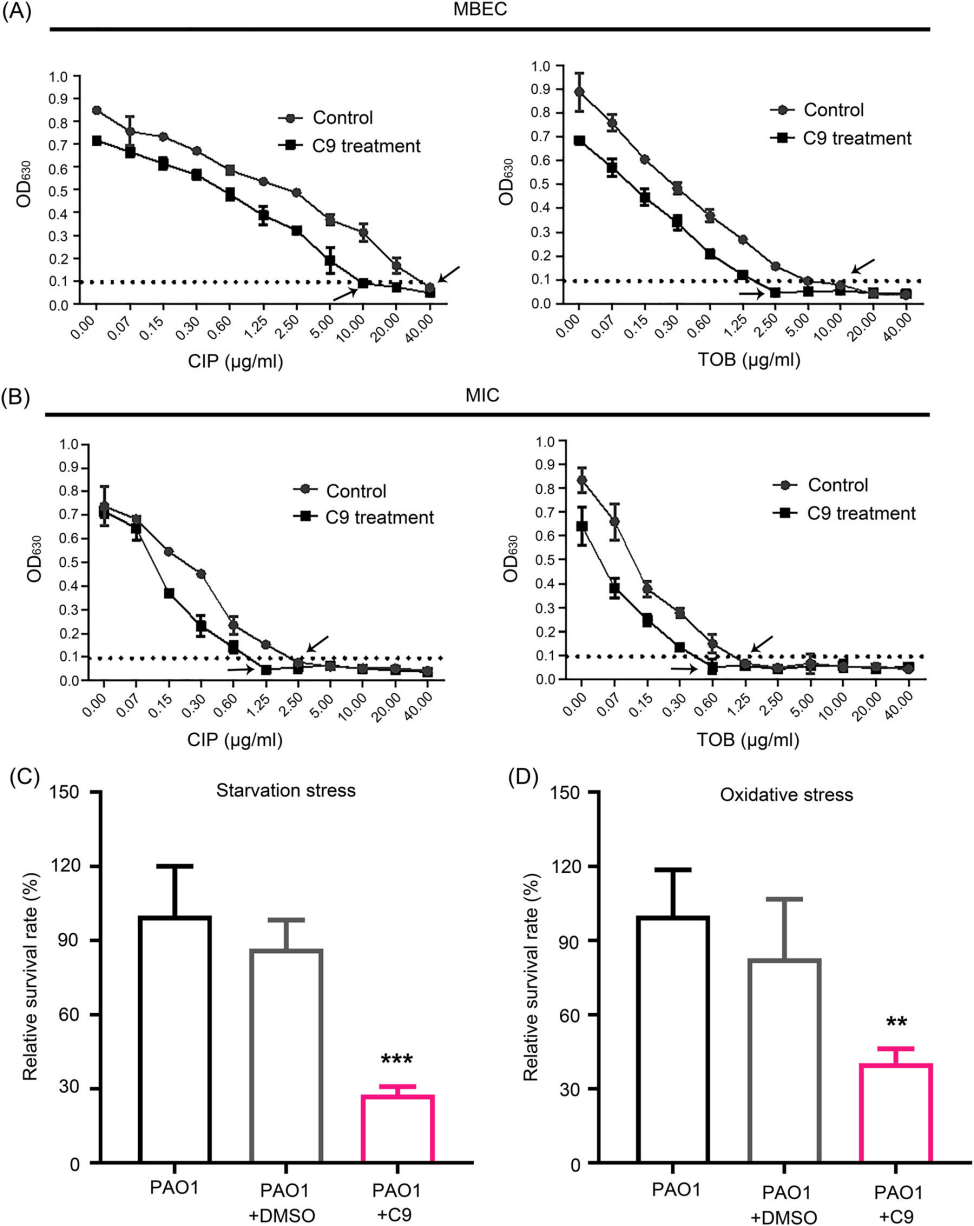
C9 as a potential antibacterial drug. Minimum biofilm eradication concentration (MBEC) (A) and minimum inhibition concentration (MIC) (B) of biofilms treated with (■) or without (●) C9. Survival rate of *Pseudomonas aeruginosa* under starvation stress (C) and oxidative stress (D) in the presence of DMSO or C9 are shown. PBS and 2 mM H_2_O_2_ were used for starvation or oxidative stress, respectively. CFUs were measured after incubation at 30°C for 6 h, and the survival rate was normalized to the level of PAO1 without treatment. The arrows indicate the value of MIC or MBEC. Error bars indicate standard deviations; significance was determined using Student's *t*‐test compared with PAO1 (***p < 0.001, **p < 0.01). CIP, ciprofloxacin; TOB, tobramycin.

The stability and low cytotoxicity of C9 indicated its excellent potential for use as an anti‐QS and antibiofilm drug. We then tested whether treatment with C9 can improve the sensitivity of the bacteria in biofilms to antibiotics by determining the minimum biofilm eradication concentration (MBEC). The Calgary Biofilm Device (CBD) was used to determine the MBEC of two selected antibiotics commonly used to treat *P. aeruginosa* infection: tobramycin (TOB) and ciprofloxacin (CIP). The MBEC of *P. aeruginosa* biofilms exposed to C9 was 4‐fold (TOB and CIP) lower than those of the no‐treatment controls (Figure 4A). Furthermore, the minimum inhibition concentration (MIC) of the two antibiotics against planktonic growth from peg‐attached biofilms was also determined. The MIC of bacteria from C9‐treated biofilm was 2‐fold lower than that of untreated biofilms (Figure 4B). Thus, treatment with C9 can sensitize bacteria in *P. aeruginosa* biofilms to antibiotics.

Previous studies have reported that the nutrient starvation response might enhance antibiotic resistance^37^, ^38^. To evaluate whether C9 could be a promising alternative against *P. aeruginosa* infections under these conditions, survivability tests of *P. aeruginosa* under starvation and oxidative stress were performed. Results showed that under starvation and oxidative stress conditions, the addition of C9 could significantly reduce the survival rates of PAO1 by approximately 70% and 60%, respectively (Figure 4C,D).

## DISCUSSION

**Figure 5:**
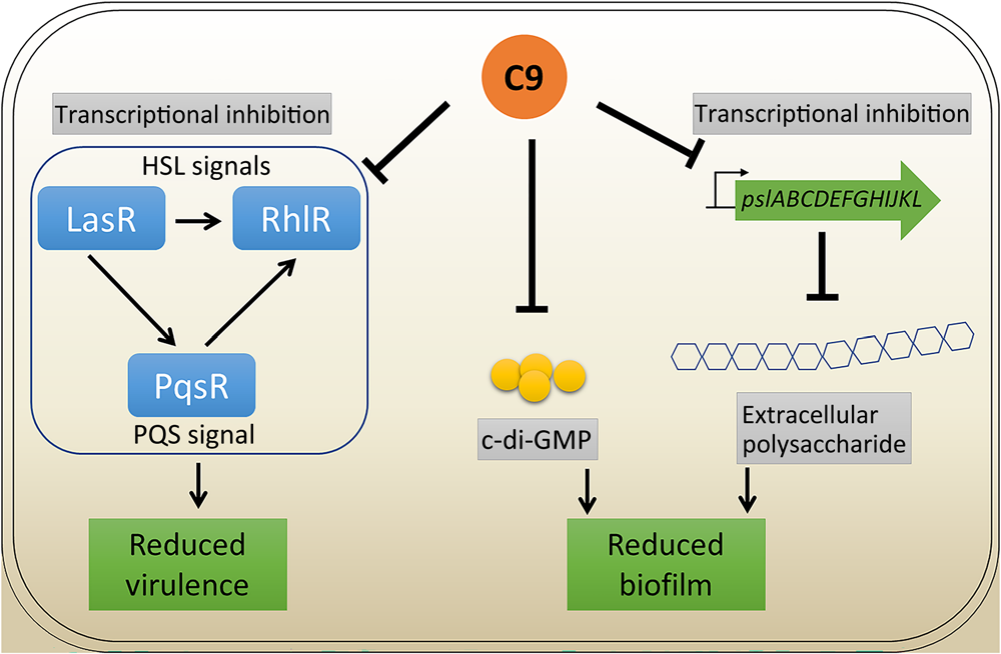
A schematic illustration showing how C9 inhibits biofilm formation and virulence in *Pseudomonas aeruginosa*. C9 inhibits biofilm formation by repressing the transcription of both HSL‐ and PQS‐related genes as well as the exopolysaccharide *psl* operon and also reduces the production of c‐di‐GMP.

Bacterial antibiotic resistance has become one of the most serious threats to human health. We are facing a growing shortage of effective antibiotics, especially for the treatment of Gram‐negative bacteria. Bacteria in biofilms are tolerant to almost all traditional antibiotics, and the EPS matrix plays a very critical role in the biofilm resistance phenotype. It plays an important role in weakening the resistance of pathogens and enabling sensitization to antibiotics, especially sensitizing of their biofilms to antibiotics. In addition, drugs that can reduce the virulence of pathogens are very effective in controlling cytokine storm during infections. Many publications have reported antibiofilm compounds, and yet, few of them targeted the EPS matrix and the virulence inhibition activity. *P. aeruginosa, E. coli*, and *S. aureus* are three common pathogens that cause nosocomial infections and often show high resistance to antimicrobial therapy due to their ability to form biofilms. Here, we report a coumarin–chalcone conjugate C9, which can inhibit the biofilm formation of these three common pathogens, with the best antibiofilm activity against *P. aeruginosa* by targeting the EPS matrix exopolysaccharide and intracellular second messenger molecule c‐di‐GMP. More importantly, C9 can significantly reduce the virulence of *P. aeruginosa*, an important Gram‐negative bacterium that causes many types of infections (Figure 5).

Coumarin derivatives have been reported to possess antibacterial^39^, antiviral^40^, antituberculosis^41^, anti‐inflammatory^42^, antioxidant^43^, anticancer^29^, anti‐QS, and antibiofilm activities^32^. Chalcones are known to have remarkable biological activities such as antibacterial, antiviral, antifungal, antimalarial, and anti‐HIV, and their synthetic derivatives have significant cytotoxic activity against various cancer cells^44^, ^45^, ^46^, ^47^, ^48^. In this study, we synthesized coumarin–chalcone conjugates C1–C12 and examined their antibiofilm activities. Among them, C9 showed the best inhibitory effect on biofilm formation, possibly due to the position of methoxy on the benzene ring. It has been reported that methoxy might be an active site responsible for the inhibition of bacterial biofilm formation^49^. Although similar or stronger biofilm‐inhibitory effects were also reported in several previous studies on other coumarin or chalcone conjugates, the effective working concentration of C9 (5 mM equal to 1.53 µg/ml) was found to be much lower than that of reported coumarin (250 µg/ml)^50^ or chalcone conjugates (100 µg/ml)^33^. Furthermore, C9 does not affect bacterial growth, indicating that it can work as a sensitizer to antibiotics. Consistently, we have shown that treatment of C9 significantly decreases the MBEC and MIC of two antibiotics, and it can also reduce the bacterial survival rates under starvation and oxidative stress conditions, indicating its potential to combat the infections caused by antibiotic‐resistant bacteria.

Exopolysaccharide is a critical matrix component of biofilms and is required for bacterial cells to adhere to a substratum for maintenance of biofilm structure and protection from antibiotics^51^, ^52^. Psl is one primary matrix exopolysaccharide of biofilms formed by *P. aeruginosa*. We have shown that the conjugate C9 inhibits the biofilm formation of *P. aeruginosa* by targeting the exopolysaccharide Psl, especially decreasing the Psl synthesis at the transcriptional level. Meanwhile, C9 can also inhibit the synthesis of c‐di‐GMP, a universal bacterial second messenger that controls exopolysaccharide synthesis and biofilm formation. Further investigations reveal that C9 can reduce the transcription of eight genes involved in the synthesis of c‐di‐GMP in *P. aeruginosa*. These data indicate that the antibiofilm activity of C9 might be attributed to its inhibitory effect on the production of Psl and c‐di‐GMP.

A previous publication has shown that the virulence of *P. aeruginosa* can be enhanced by decreasing the c‐di‐GMP level^53^. Strikingly, C9 reduces the c‐di‐GMP synthesis, and yet, it can also significantly reduce the virulence of *P. aeruginosa* both in vivo and in vitro by inhibiting the transcription of several QS regulators, especially interfering with the function of LasR and PqsR, which are two key QS regulators. Consequently, the antagonistic activity of conjugate C9 could be attributed to its capacity to obstruct the natural ligands of QS signal molecules to the binding site on the QS receptor proteins. LasR has been reported to activate the transcription of the *psl* operon^54^. Thus, the reduction of C9 on Psl production could be a result of repression of LasR. Overall, C9 inhibits biofilm formation and reduces the virulence of *P. aeruginosa* by repressing c‐di‐GMP, Psl production, and the QS system, as summarized in Figure 5.

Novel antibiofilm and/or anti‐QS compounds are urgently needed. As many antibiofilm compounds were found to increase virulence^55^, compounds that can inhibit biofilm and reduce virulence are thus very useful and have excellent potential for use as antibiofilm drugs. In the present study, we demonstrated that the coumarin–chalcone conjugate C9 could not only inhibit the biofilm formation of *P. aeruginosa* by reducing the production of Psl and c‐di‐GMP but it could also reduce bacterial virulence by affecting the transcription of QS genes. Besides, C9 treatment markedly enhances the biofilm sensitivity to antibiotics and can also reduce the survival rates of PAO1 under starvation and oxidative stress conditions. Moreover, C9 shows excellent stability at a range of pH values as well as antibiofilm activity against two other bacteria related to hospital‐acquired infections: *S. aureus* and *E. coli*. Overall, our study indicates that the coumarin–chalcone conjugate C9 has excellent potential for use as a broad‐spectrum agent to combat biofilm‐related complications.

## MATERIALS AND METHODS

### Bacterial strains and growth conditions

Luria Bertani (LB) was used for *P. aeruginosa*, Nutrient broth (NB) was used for *E. coli*, and Tryptic Soy Broth (TSB) was used for *S. aureus*. Strains were grown under aeration at 37°C. When necessary, for selection or to maintain plasmids, cultures were supplemented with 300 μg/ml carbenicillin for *P. aeruginosa* or 100 μg/ml ampicillin for *E. coli*.

### Synthesis of coumarin–chalcone conjugates

Substituted coumarin–chalcone derivatives (C1–C12) were synthesized by the Claisen–Schmidt condensation reaction as previously described^56^, ^57^. In brief, equivalent amounts of 3‐acetyl coumarin (0.01 mol) and substituted benzaldehyde (0.01 mol) were taken in a 50 ml single‐neck round‐bottom flask. Then, 10 ml of ethanol was added to the reaction mixture (R.M.) and stirred for 5 min. Later, two to three drops of piperidine were added to R.M. and refluxed for 12–15 h. The progress of the reaction was examined by thin‐layer chromatography (TLC). After completion of the reaction, the reaction mixture was quenched in crushed ice; the solid product was filtered, then washed with water, and recrystallized from ethanol. All solvents and chemicals were used as purchased without further purification. The starting material 3‐acetyl coumarin was purchased from Merck. The progress of all reactions was monitored on precoated silica gel plates (Merck, with fluorescence indicator UV254) using the Hexane:ethyl acetate (70:30) system as the mobile phase. Spots were visualized by irradiation with ultraviolet light (254 nm). The structure of compounds was confirmed by the NMR spectrum.

### pH titrimetric assay

Stock solutions of compound C9 (0.5 mM), HCl, and KOH (25 mM each) were prepared in an acetonitrile and water mixture (8:2). The pKa of the compounds was measured by potentiometric titration experiments^58^, ^59^ using a pH meter (HI5000 Series; Hanna), which was calibrated with standard buffers of pH 4 and 7. The initial pH of compound C9 was found to be 6.9 and this pH was gradually decreased to about pH 2 by slowly titrating with HCl (25 mM). After reaching pH 2, the compound was titrated with the slow addition of KOH (25 mM) till the pH reached 12. The solution stability of C9 under varied pH conditions^59^ was determined using a UV‐1800 spectrophotometer from Shimadzu.

### Growth curve analysis

Overnight culture of *P. aeruginosa*, *E. coli*, and *S. aureus* strains was diluted with fresh medium (1:100). The bacterial culture was then incubated with or without coumarin–chalcone C9 (5 mM) at 37°C, 200 rpm for 24 h, and OD_600_ was determined hourly to trace bacterial growth using a Synergy H4 hybrid reader (BioTek). Additionally, the effects of C9 on the bacterial growth of *P. aeruginosa* strain, *E. coli*, and *S. aureus* were measured using a dis‐diffusion assay on plates.

### Biofilm assay and confocal microscopy

The biofilm assay was performed as previously described^60^. Briefly, 1% of overnight bacterial culture was inoculated in Jensen's medium with 5 mM coumarin–chalcone C1–C9 or chalcone (Acmec) or 3‐acetyl coumarin (Acmec) and grown in triplicate in a polyvinyl chloride plate (costar) overnight at 30°C without agitation, followed by staining with 0.1% crystal violet for 30 min at room temperature. The adherent stain was solubilized in 30% acetic acid and OD_560_ was determined to quantify the biofilm biomass. Air–liquid interface biofilms (pellicles) were grown in glass chambers (Chambered #1.5 German Coverglass System, Nunc) with a glass coverslip at the bottom of each chamber at 30°C for 24 h. One milliliter of a 1/100 dilution of *P. aeruginosa* overnight culture in Jensen's medium was inoculated into the chamber. The biofilms were stained with DNA stain SYTO9 (Molecular PROBEs; Invitrogen) for 15 min (5 µM concentration, molecular PROBE; Invitrogen) and then assessed using an FV1000 CLSM (Olympus) with the 63×/1.3 objective at 488 nm (SYTO9). The CLSM images were processed using COMSTAT software^61^.

### Immunodot blotting of Psl polysaccharide

Psl immunoblots were performed as previously described, with minor changes^62^. *P. aeruginosa* PAO1 cells were incubated in Jensen's medium with shaking at 37°C for 24 h and treated with 5 mM C9 or DMSO as a control. Culture with an OD_600_ of 4 was centrifuged and the pellet was suspended in 100 µl of 0.5 M EDTA; extracts were boiled at 100°C for 5 min. The supernatant was treated with proteinase K for 1 h (0.5 mg/ml) at 60°C, followed by treatment at 80°C for 30 min to inactivate proteinase K. Three microliters of polysaccharide preparations were spotted onto a nitrocellulose membrane. Blocking was performed with 10% skim milk in TBST (20 mM Tris, 137 mM NaCl, 0.1% Tween 20 [pH 7.6]) at room temperature for 1 h. The anti‐Psl antibodies (1:25,000 dilution) and 1:7500‐diluted goat antirabbit IgG‐conjugated secondary antibody were used for the detection of Psl. The immunoblotting data were analyzed using Image Lab software. At least three technical replicates were performed for each sample.

### Measurement of β‐galactosidase activity

β‐Galactosidase activity was measured as described in a previous study^63^. Overnight *P. aeruginosa* cells were diluted (1:100) in Jensen's media with or without Coumarin–chalcone C9, incubated at 37°C, and then 5 ml of culture (OD_600_ about 0.5) was harvested. β‐Galactosidase activity of cell lysates was measured and the results were expressed in Miller units. Experiments were carried out in triplicate.

### Measurement of fluorescence intensity

Table S1 shows the information for the plasmids constructed in this study. For promoter–*gfp* transcriptional fusions, the vector pPROBE‐AT′ (Ap^r^) was utilized^64^. The intergenic region was based on the *Pseudomonas* Genome Database (https://www.pseudomonas.com). The software predicting the promoter regions and RBS (ribosome binding site) is in the following websites (http://www.phisite.org/main/index.php?nav=tools&navsel=hunter, https://services.healthtech.dtu.dk/service.php?Promoter-2.0 and https://www.fruitfly.org/seq_tools/promoter.html)^65^, ^66^, ^67^. The corresponding plasmid (Table S1) was then transformed into *P. aeruginosa* to determine the transcription level of the corresponding genes. To determine the fluorescence intensity of the promoter, the overnight cultures of the plasmid‐carrying strains grown in Jensen's medium with carbenicillin were inoculated at 1% into 200 μl of Jensen's medium containing 5 mM C9 or DMSO in 96‐well plates and grown by shaking at 700 rpm in a constant‐temperature microplate shaker (MIULAB Co.) at 37°C for 24 h. The fluorescence of GFP was measured every 4 h for 24 h using a Synergy H4 hybrid reader (BioTek) at an excitation wavelength of 488 nm and an emission wavelength of 520 nm with gain of 50. To account for the background fluorescence, the fluorescence from pPROBE‐AT′ was subtracted after the fluorescence signal value was normalized to OD_600_. All experiments were performed with a minimum of three biological replicates.

### Analysis of intracellular c‐di‐GMP

Indirect c‐di‐GMP was analyzed using the pCdrA::*gfp* ^s^ plasmid (Table S1), in which the c‐di‐GMP‐dependent *cdrA* promoter is fused to GFP^68^. Direct intracellular c‐di‐GMP was measured by LC‐MS/MS as previously described^69^.

### Isolation of total RNA and the qRT‐PCR assay

*P. aeruginosa* was cultivated in Jensen's medium with or without C9 treatments (5 mM) at 37°C, 200 rpm, until OD_600_ reached 3.0. Bacteria were harvested by centrifugation and RNA samples were extracted using the RNAprep pure bacterial kit (Tiangen Co.). Total cDNA was generated using the HiScript III all‐in‐one RT SuperMix Perfect for qPCR (Vazyme Co.). The ChamQ universal SYBR qPCR master mix (Vazyme Co.) and gene‐specific primers listed in Table S2 were used to perform qRT‐PCR in a real‐time PCR system (Applied Biosystems ViiA 7). The experiments were repeated at least three times. Real‐time PCR data were normalized to *rpsL* transcripts and calculated according to the instructions of the manufacturer.

### LC‐MS/MS analysis

The three QS signaling molecules of 3‐oxo‐C_12_‐HSL, C_4_‐HSL, and PQS were quantified using LC‐MS/MS as described^70^. Briefly, *P. aeruginosa* strains with 5 mM C9 treatment or DMSO as a control were grown in LB media at 37°C for 24 h. One milliliter of bacterial cultures was spun down and supernatants were collected for LC‐MS/MS analysis using a mass spectrometer (QTRAP 6500 System; AB SCIEX) for further determination. The experiments were repeated at least three times. N‐(3‐oxododecanoyl)‐l‐homoserine lactone (3‐oxo‐C_12_‐HSL), N‐butanoyl‐l‐homoserine lactone (C_4_‐HSL), and 2‐heptyl‐3‐hydroxy‐4‐quinolone (PQS) were used as standards for the calculation of the QS signal molecule concentrations.

### Virulence test in a plant model

The virulence test was carried out according to a previously described method^71^. Briefly, *P. aeruginosa* strains with different treatments were grown overnight at 37°C in LB medium to reach a stationary phase. Bacterial cells were collected by centrifugation, then washed and diluted to around 10^8^ CFU ml^−1^ in 10 mM MgSO_4_, and 10 μl of these bacterial suspensions were injected using a syringe into the midrib of Chinese cabbage leaves that had been previously washed with 5% bleach. The leaves were placed on new petri dishes with MgSO_4_‐impregnated Whatman filters. The plates were kept in a growth chamber at 37°C and rot symptoms were monitored for 3 days. The experiments were repeated at least three times.

### Pyocyanin production assay

Pyocyanin production was determined on the basis of the absorbance of pyocyanin at 520 nm in an acidic solution. Five mililiters of overnight bacterial supernatant with or without C9 (5 mM) was mixed with 3 ml of chloroform and pyocyanin in the chloroform phase was then re‐extracted by adding 1 ml of 0.2 N HCl, showing a pink to deep red solution. The OD_520_ values of these corresponding solutions were measured, and bacterial density was also recorded by measurement of OD_600_. The ratio of OD_520_/OD_600_ was used for pyocyanin production.

### Motility assay

Overnight culture from the plate was spotted and stabbed on a swim agar (tryptone‐10 g/l, yeast extract‐ 5 g/l, agar‐0.3%) cultivated at 37°C overnight^72^. Twitching motility was assayed by stabbing inoculated strains through a thin LBNS media agar (tryptone‐10 g/l, yeast extract‐5 g/l, agar‐1%) plate, followed by incubation at 30°C for 24 h under humidified conditions. Twitching zones were visualized at the agar–plate interface^73^.

### Detection of the MIC and the MBEC

The MIC and MEBC values of the biofilm formed on the CGM, CBD cartridge were determined according to the method of the MBEC™ High‐throughput Experimental Manual (Innovotech) as described^74^. Briefly, biofilms were formed on plastic pegs on the lid of the CBD, and the Peg‐biofilms were exposed to media with or without 5 mM C9 before antibiotic challenge with TOB (Tobramycin) or CIP (Ciprofloxacin). The MIC value represents the minimum concentration of antibiotic required to inhibit the growth of free bacteria from Peg‐biofilms (OD_600_ < 0.1). MBEC was measured by ultrasonic disruption of the biofilm after treatment with C9 and continuous culture at 37°C for 24 h.

### Survival rate assay

The survival rate of PAO1 under starvation and oxidative stress was determined as previously described, with slight modifications^38^, ^75^. The overnight bacterial culture was centrifuged, and the supernatant was discarded and then resuspended in the corresponding medium separately according to different severe conditions. PBS or LB with 2 mM H_2_O_2_ was used for the survival rate test under starvation or oxidative stress, with C9 (5 mM) or DMSO added to the experimental or control groups, and the CFUs were measured after incubation at 30°C for 6 h.

### Molecular docking analysis

The docking analysis for C9 and proteins was performed using the Swissdock server (www.swissdock.ch) with default parameters according to the reported protocol^71^. The molecular docking scores and 2D/3D interaction images were predicted.

### Macrophage cytotoxicity assay

Cytotoxicity of C9 against macrophages was determined by the MTT assay using the MTT Cell Proliferation Cytotoxicity assay kit (Solarbio). Briefly, exponentially growing J774A.1 macrophages were seeded in 96‐well microtiter plates and incubated at 37°C, 5% CO_2_, for 24 h. Then, the medium was replaced with fresh DMEM medium, and C9 of various concentrations or DMSO as a control were added, followed by a 24‐h incubation. After incubation with MTT for 4 h at 37°C, the formazan solution was added and cell viability was monitored by the measurement of OD_490_. Each experiment was performed with three independent replicates.

## AUTHOR CONTRIBUTIONS

**Yu Zhang**: Data curation (equal); formal analysis (equal); investigation (equal); writing—review and editing (equal). **Pramod Bhasme**: Investigation; writing—original draft. **Dinesh S. Reddy**: Investigation; writing—original draft. **Dejian Liu**: Investigation; writing—review and editing. **Zhaoxiao Yu**: Investigation; writing—review and editing. **Tianhu Zhao**: Investigation. **Yaqian Zheng**: Investigation. **Amit Kumar**: Methodology (lead); writing—original draft. **Haiying Yu**: Funding acquisition (equal); methodology (supporting); project administration (equal). **Luyan Z. Ma**: Conceptualization (lead); data curation (lead); formal analysis (lead); funding acquisition (lead); resources (lead); supervision (lead); validation (lead); visualization (lead); writing—original draft (lead); writing—review and editing (lead).

## ETHICS STATEMENT

This study did not conduct animal or human experiments. There are no ethical issues involved.

## CONFLICT OF INTERESTS

The authors declare no conflict of interests.

## Supporting information


**Figure S1. NMR spectra of compound (C9)**. (A) ^1^H NMR spectra (B) ^13^C NMR spectra.

**Figure S2. Effects of coumarin–chalcone conjugates on the bacterial biofilm, motilities, and growth**. (A) Inhibitory effect of coumarin and chalcone on the *P. aeruginosa* strain PAO1 biofilm. (B‐D) Effects of C9 (5 mM) on established biofilms of PAO1, its swimming motility, and twitching motility. (E) Effects of C9 (5 mM) on the bacterial growth of PAO1, *E. coli*, and *S. aureus* in LB and the disc‐diffusion assay on plates. Error bars indicate standard deviations, highly significant difference (**, *P* < 0.01) compared with controls.

**Figure S3. Interaction diagram of compound C9 ligand with (A)** ***lasR*** and ***(B) pqsR*** **genes**. Interaction diagram of the C9 ligand with LasR, PqsR and The yellow dotted lines in 3D figure show the hydrogen bond between the ligand and protein receptor, and the 2D interaction diagram shows C9 interaction with both genes; one hydrogen bond is between the ligand and the 129^th^ Serine residue in the receptor of protein LasR. The hydrogen bonding between C9 and PqsR is with the 62^nd^ Tryptophan residue of protein.

**Table S1**. Plasmids used in this study.
**Table S2**. Primers for qRT‐PCR used in this study.

## Data Availability

All data generated or analyzed during this study are included in this article.
