# Supplementary figures and images for: Dual functions: A coumarin–chalcone conjugate inhibits cyclic‐di‐GMP and quorum‐sensing signaling to reduce biofilm formation and virulence of pathogens
Source: mLife. 2023 Sep 24;2(3):283–94. doi: 10.1002/mlf2.12087 (PMC10989777; doi:10.1002/mlf2.12087)

(A)

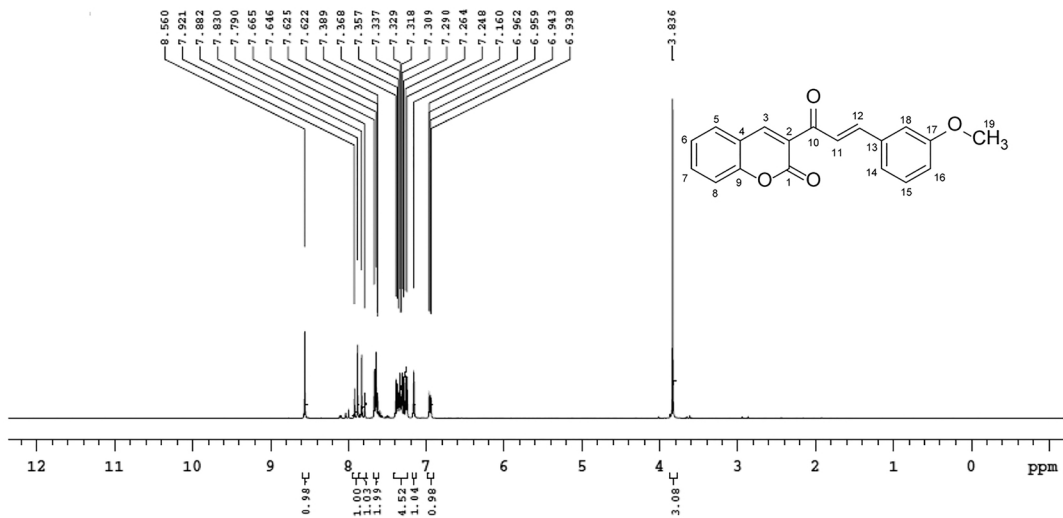

(B)

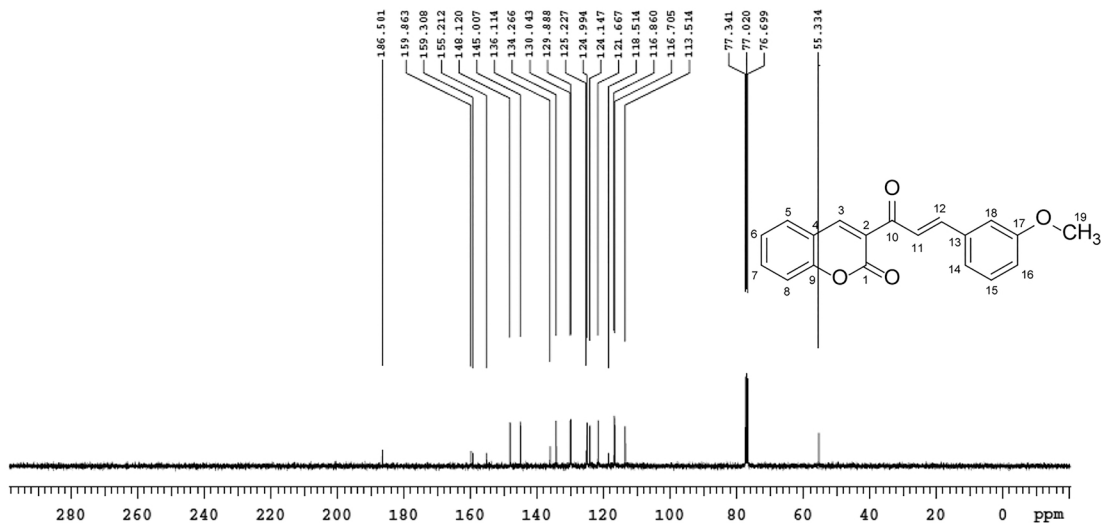

Supplement: Supplementary file 1 — Figure S1. NMR spectra of compound (C9). (A) 1H NMR spectra (B) 13C NMR spectra. [file MLF2-2-283-s002.pdf]

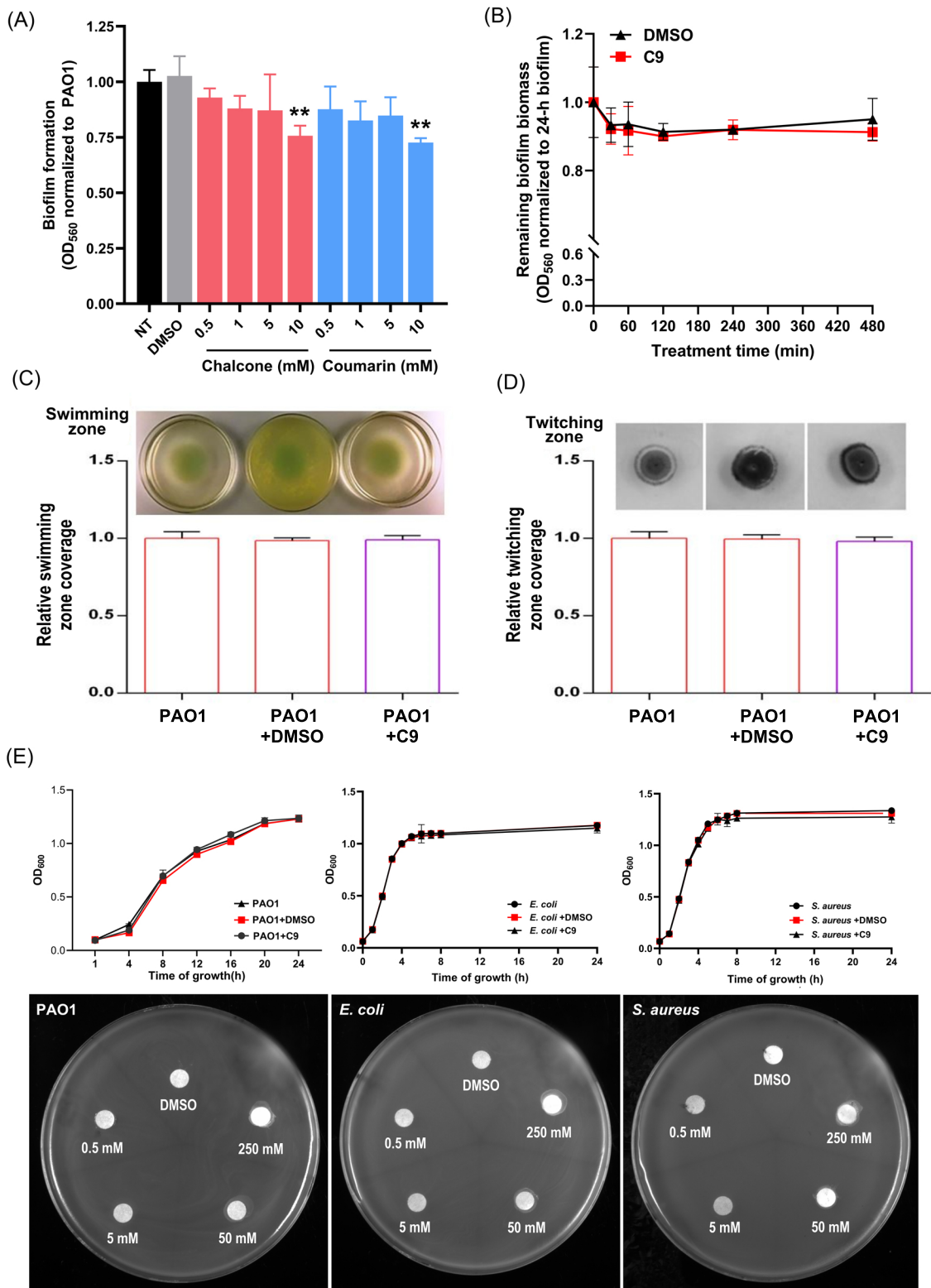

Supplement: Supplementary file 2 — Figure S2. Effects of coumarin–chalcone conjugates on the bacterial biofilm, motilities, and growth. (A) Inhibitory effect of coumarin and chalcone on the P. aeruginosa strain PAO1 biofilm. (B‐D) Effects of C9 (5 mM) on established biofilms of PAO1, its swimming motility, and twitching motility. (E) Effects of C9 (5 mM) on the bacterial growth of PAO1, E. coli, and S. aureus in LB and the disc‐diffusion assay on plates. Error bars indicate standard deviations, highly significant difference (**, P < 0.01) compared with controls. [file MLF2-2-283-s001.pdf]

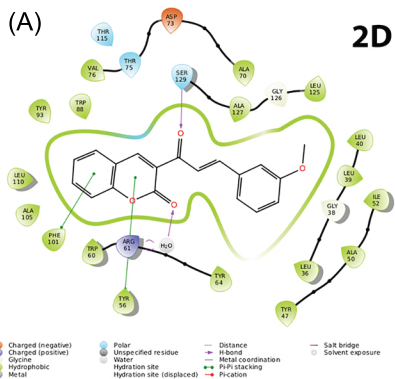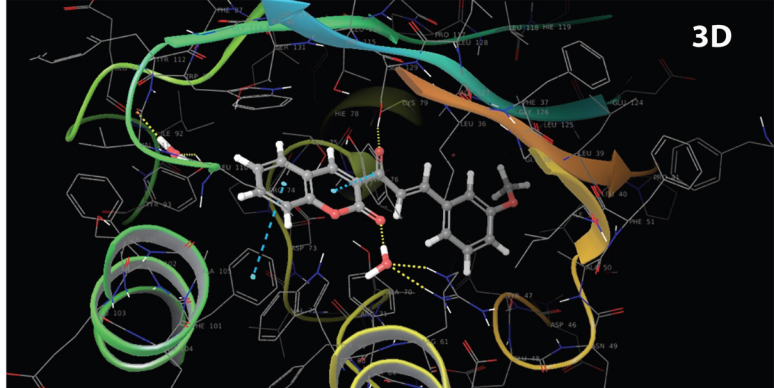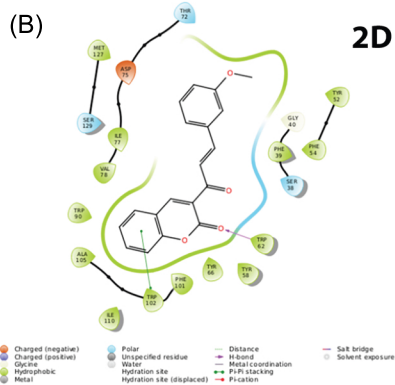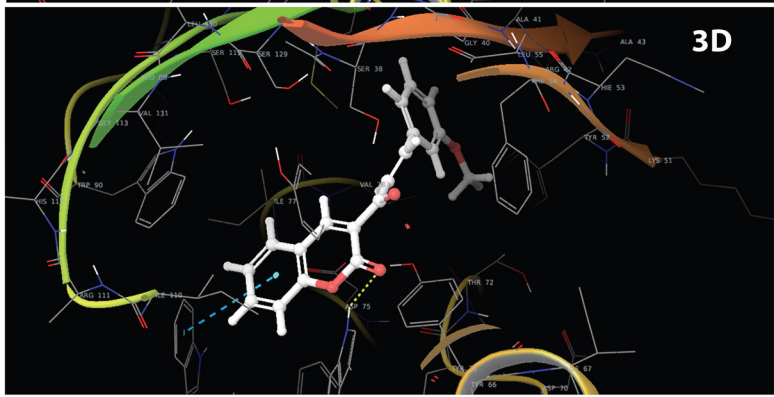

Supplement: Supplementary file 3 — Figure S3. Interaction diagram of compound C9 ligand with (A) lasR and (B) pqsR genes. Interaction diagram of the C9 ligand with LasR, PqsR and The yellow dotted lines in 3D figure show the hydrogen bond between the ligand and protein receptor, and the 2D interaction diagram shows C9 interaction with both genes; one hydrogen bond is between the ligand and the 129th Serine residue in the receptor of protein LasR. The hydrogen bonding between C9 and PqsR is with the 62nd Tryptophan residue of protein. [file MLF2-2-283-s004.pdf]
