# Supplementary material for: Dual functions: A coumarin–chalcone conjugate inhibits cyclic‐di‐GMP and quorum‐sensing signaling to reduce biofilm formation and virulence of pathogens
Source: mLife. 2023 Sep 24;2(3):283–94. doi: 10.1002/mlf2.12087 (PMC10989777; doi:10.1002/mlf2.12087)
Supplement: Supplementary file 4 — Table S1. Plasmids used in this study. Table S2. Primers for qRT‐PCR used in this study. [file MLF2-2-283-s003.docx]

**Supporting Information**

**Table S1.** Plasmids used in this study

| **Plasmid** | **Description** | **Source** |
| --- | --- | --- |
| pPROBE-AT′ | Promoterless *gfp* reporter plasmid (Ap^r^) | (64) |
| pCdrA::*gfp*^s^ | pUCP22Not-PcdrA-CDS-RNaseIII-*gfp*-T0-T1, Amp^r^ Gm^r^ | (68) |
| pTH1 | *pslA*::*lacZ* in mimi-CTX *lacZ*, Tc^r^ | In this study |
| pTH2 | *lasR*::*gfp* in pPROBE-AT', Ap^r^ | In this study |
| pTH3 | *rhlR*::*gfp* in pPROBE-AT', Ap^r^ | In this study |
| pTH4 | *pqsR*::*gfp* in pPROBE-AT', Ap^r^ | In this study |
| pTH5 | *pqsA*::*gfp* in pPROBE-AT', Ap^r^ | In this study |
| pPA0169 | Promoter sequence of PA0169 inserted into pPROBE-AT′ (Ap^r^) | In this study |
| pPA0290 | Promoter sequence of PA0290 inserted into pPROBE-AT′ (Ap^r^) | In this study |
| pPA0338 | Promoter sequence of PA0338 inserted into pPROBE-AT′ (Ap^r^) | In this study |
| pPA0847 | Promoter sequence of PA0847 inserted into pPROBE-AT′ (Ap^r^) | In this study |
| pPA1107 | Promoter sequence of PA1107 inserted into pPROBE-AT′ (Ap^r^) | In this study |
| pPA1120 | Promoter sequence of PA1120 inserted into pPROBE-AT′ (Ap^r^) | In this study |
| pPA1851 | Promoter sequence of PA1851 inserted into pPROBE-AT′ (Ap^r^) | In this study |
| pPA2771 | Promoter sequence of PA2771 inserted into pPROBE-AT′ (Ap^r^) | In this study |
| pPA2870 | Promoter sequence of PA2870 inserted into pPROBE-AT′ (Ap^r^) | In this study |
| pPA3177 | Promoter sequence of PA3177 inserted into pPROBE-AT′ (Ap^r^) | In this study |
| pPA3343 | Promoter sequence of PA3343 inserted into pPROBE-AT′ (Ap^r^) | In this study |
| pPA3702 | Promoter sequence of PA3702 inserted into pPROBE-AT′ (Ap^r^) | In this study |
| pPA4332 | Promoter sequence of PA4332 inserted into pPROBE-AT′ (Ap^r^) | In this study |
| pPA4396 | Promoter sequence of PA4396 inserted into pPROBE-AT′ (Ap^r^) | In this study |
| pPA4843 | Promoter sequence of PA4843inserted into pPROBE-AT′ (Ap^r^) | In this study |
| pPA4929 | Promoter sequence of PA4929 inserted into pPROBE-AT′ (Ap^r^) | In this study |
| pPA5487 | Promoter sequence of PA5487 inserted into pPROBE-AT′ (Ap^r^) | In this study |

**Table S2**. Primers for qRT-PCR used in this study

| **Primer** | **Sequence (5’→3’)** |
| --- | --- |
| RT-PA4843-F | ACAAAGGCACCCACCAACAG |
| RT-PA4843-R | GGTCATCGTGCTCGGTCATC |
| RT-PA4396-F | AAATCGGCTTCGGCGGTGAG |
| RT-PA4396-R | CCTTCGTTCGACGTGGTGTCC |
| RT-PA1107-F | AGGCGGCTGCCGGAATGAAA |
| RT-PA1107-R | CACCTCGATGGCGTGCTGGA |
| RT-PA4929-F | TACTTGGGATAGTCGAACAGCG |
| RT-PA4929-R | GTATAGAAAGAAGGTGGCGAAATG |
| RT-PA3343-F | GACGCCAAGCCCTCCAAGAG |
| RT-PA3343-R | TCGGGAAGGAAACGCAGACC |
| RT-PA0290-F | TACGAACGGTATCGCTCGGCAACT |
| RT-PA0290-R | TCCTGCGGGTCTTCGGGAAT |
| RT-PA5487-F | CCTGCCCTGTTCGGCGGTAT |
| RT-PA5487-R | TTCCTTGTCCACGCCCTTGC |
| RT-PA0847-F | CGCTCGCCTTCATGCCTTTC |
| RT-PA0847-R | CCTTGCGTGCTTCGTTCCTG |
| RT-*lasI*-F | GGTCCGCACCTGAAGATC |
| RT-*lasI*-R | TGTTCCACCAGCACTCCC |
| RT-*lasR*-F | GCTGGAACGCTCAAGTGG |
| RT-*lasR*-R | AACAGGCCGAACAGGATC |
| RT-*rhlI*-F | CTGGTCCAGCCTGCAATG |
| RT-*rhlI*-R | TGGAGGATCACGCCGTTG |
| RT-*rhlR*-F | TCCGATGCTGATGTCCAA |
| RT-*rhlR*-R | TCAGGATGATGGCGATTT |
| RT-*pqsA*-F | CGCCGAACAGATTCCCTC |
| RT-*pqsA*-R | CAACATGCCCGTTCCTCC |
| RT-*pqsR*-F | AGCCTGATACGCACCTCG |
| RT-*pqsR*-R | CACTGGTTGAAGCGGGAG |
